# Quantification of bone marrow lesion volume and volume change using semi-automated segmentation: data from the osteoarthritis initiative

**DOI:** 10.1186/1471-2474-14-3

**Published:** 2013-01-02

**Authors:** Jincheng Pang, Jeffrey B Driban, Geoffroy Destenaves, Eric Miller, Grace H Lo, Robert J Ward, Lori Lyn Price, John A Lynch, Charles B Eaton, Felix Eckstein, Timothy E McAlindon

**Affiliations:** 1 Department of Electrical and Computer Engineering, Tufts University, 216 Halligan Hall, Medford, MA, 02155, USA; 2 Division of Rheumatology, Tufts Medical Center, 800 Washington Street, Box #406, Boston, MA, 02111, USA; 3 Medical Care Line and Research Care Line; Houston Health Services Research and Development (HSR&D) Center of Excellence Michael E. DeBakey VAMC, Houston, TX, USA; 4 Department of Radiology, Tufts Medical Center, 800 Washington Street, Box #299, Boston, MA, 02111, USA; 5 Biostatistics Research Center, Institute for Clinical Research and Health Policy Studies, Tufts Medical Center, 800 Washington Street, Box #63, Boston, MA, 02111, USA; 6 Department of Epidemiology and Biostatistics, University of California at San Francisco, 185 Berry Street, Lobby 5, Suite 5700, San Francisco, CA, 94107, USA; 7 Center for Primary Care and Prevention, Alpert Medical School of Brown University, Pawtucket, RI, USA; 8 Institute of Anatomy and Musculoskeletal Research, Paracelsus Medical University, Salzburg, Austria; 9 Chondrometrics GmbH, Ainring, Germany; 10 Section of Immunology, Allergy, and Rheumatology, Baylor College of Medicine, Houston, TX, USA

**Keywords:** Knee, Magnetic resonance imaging, Osteoarthritis

## Abstract

**Background:**

To determine the validity of a semi-automated segmentation of bone marrow lesions (BMLs) in the knee.

**Methods:**

Construct validity of the semi-automated BML segmentation method was explored in two studies performed using sagittal intermediate weighted, turbo spine echo, fat-suppressed magnetic resonance imaging sequences obtained from the Osteoarthritis Initiative. The first study (n = 48) evaluated whether tibia BML volume was different across Boston Leeds Osteoarthritis Knee Scores (BLOKS) for tibia BMLs (semiquantitative grades 0 to 3). In the second study (n = 40), we evaluated whether BML volume change was associated with changes in cartilage parameters. The knees in both studies were segmented by one investigator. We performed Wilcoxon signed-rank tests to determine if tibia BML volume was different between adjacent BLOKS BML scores and calculated Spearman correlation coefficients to assess the relationship between 2-year BML volume change and 2-year cartilage morphometry change (significance was p ≤ 0.05).

**Results:**

BML volume was significantly greater between BLOKS BML score 0 and 1 (*z* = 2.85, *p* = 0.004) and BLOKS BML scores 1 and 2 (*z* = 3.09, *p* = 0.002). There was no significant difference between BLOKS BML scores 2 and 3 (*z* = −0.30, *p* = 0.77). Increased tibia BML volume was significantly related to increased tibia denuded area (Spearman *r* = 0.42, *p* = 0.008), decreased tibia cartilage thickness (Spearman *r* = −0.46, *p* = 0.004), increased femur denuded area (Spearman *r* = 0.35, *p* = 0.03), and possibly decreased femur cartilage thickness (Spearman *r* = −0.30, *p* = 0.07) but this last finding was not statistically significant.

**Conclusion:**

The new, efficient, and reliable semi-automated BML segmentation method provides valid BML volume measurements that increase with greater BLOKS BML scores and confirms previous reports that BML size is associated with longitudinal cartilage loss.

## Background

Periarticular bone changes are integral to knee osteoarthritis (OA) progression [1-6]. More specifically, bone marrow lesions (BMLs), common magnetic resonance (MR) imaging findings in joints with OA, are related to OA progression and pain [1-7]. In fat suppressed MR images, BMLs are characterized as areas of high-signal intensity within bone [8,9]. Several methods can assess BML size in knees including semi-quantitative scoring [8,10], approximating BML size based on linear measurements [5,6,11,12], or manual/semi-automated segmentation [13-17].

While the current techniques have clarified the clinical relevance of BMLs in OA progression they may be limited by a lack of sensitivity to change, a dependence on the reader to identify BMLs, or the amount of time required to quantify BML size. One simple approach to measure BML size is to measure the greatest cross-sectional diameter of a BML [5]. This method is time-efficient and offers a quantitative outcome; however, only one dimension is under scrutiny; therefore, other changes may occur that go unnoticed. On the other hand, three-dimensional measurements, based on three cross-sectional measures [6,11,12], are a time efficient method to assess BMLs with good construct validity but include a lot of healthy bone. The most accurate method to assess BML size may be detailed image or volumetric segmentation [13,14] but to date accurate results require substantial user interaction and thus requires an extensive amount of time to complete. For example, semi-automated segmentation methods often use manual steps that may introduce measurement error or increase the burden on the assessor (e.g., manually delineating regions of interest, manually marking areas of health bone) [16,17]. Ideally, a new quantitative BML measurement would be accurate and time efficient.

The purpose of this study was to determine the validity of a new semi-automated BML segmentation method. We hypothesize that this new measure of BML size will increase with greater semi-quantitative scores and relate to articular cartilage loss (construct validity). This approach not only reduces the time involved in segmentation, but can also easily be deployed by researchers.

## Methods

To assess the validity of the new semi-automated BML segmentation method we conducted two analyses using images and data obtained from the Osteoarthritis Initiative (OAI) databases, which are available for public access at http://oai.epi-ucsf.org. The OAI is a multi-center observational cohort study of knee OA that collected longitudinal clinical and image data [18] as well as biospecimens over a nine year period. All BML measurements were performed using sagittal intermediate weighted, turbo spine echo, fat-suppressed MR sequences (field of view = 160 mm, slice thickness = 3 mm, skip = 0 mm, flip angle = 180 degrees, echo time = 30 ms, recovery time = 3200 ms, 313 X 448 matrix [interpolated to 512 X 512], phase encode superior/inferior. x resolution = 0.357 mm, and y resolution = 0.511 mm). All images were obtained using one of four identical Siemens Trio 3 T MR systems and a USA Instruments quadrature transmit-receive knee coil at one of four OAI clinical sites.

### Semi-automated BML segmentation

The new semi-automated segmentation method detects, extracts, and quantifies the structure of BMLs in three major steps: bone segmentation, BML segmentation, and BML quantification. In the first step, boundaries of tibia and femur were identified using a process scheme that is automatic except for some coarse initialization provided by the user. The second, fully automated step segmented BMLs within the tibia and femur regions identified from the first step. The third step which is also fully automated eliminated some false positive regions identified in the previous step and calculated the BML volumes.

**Figure 1:**
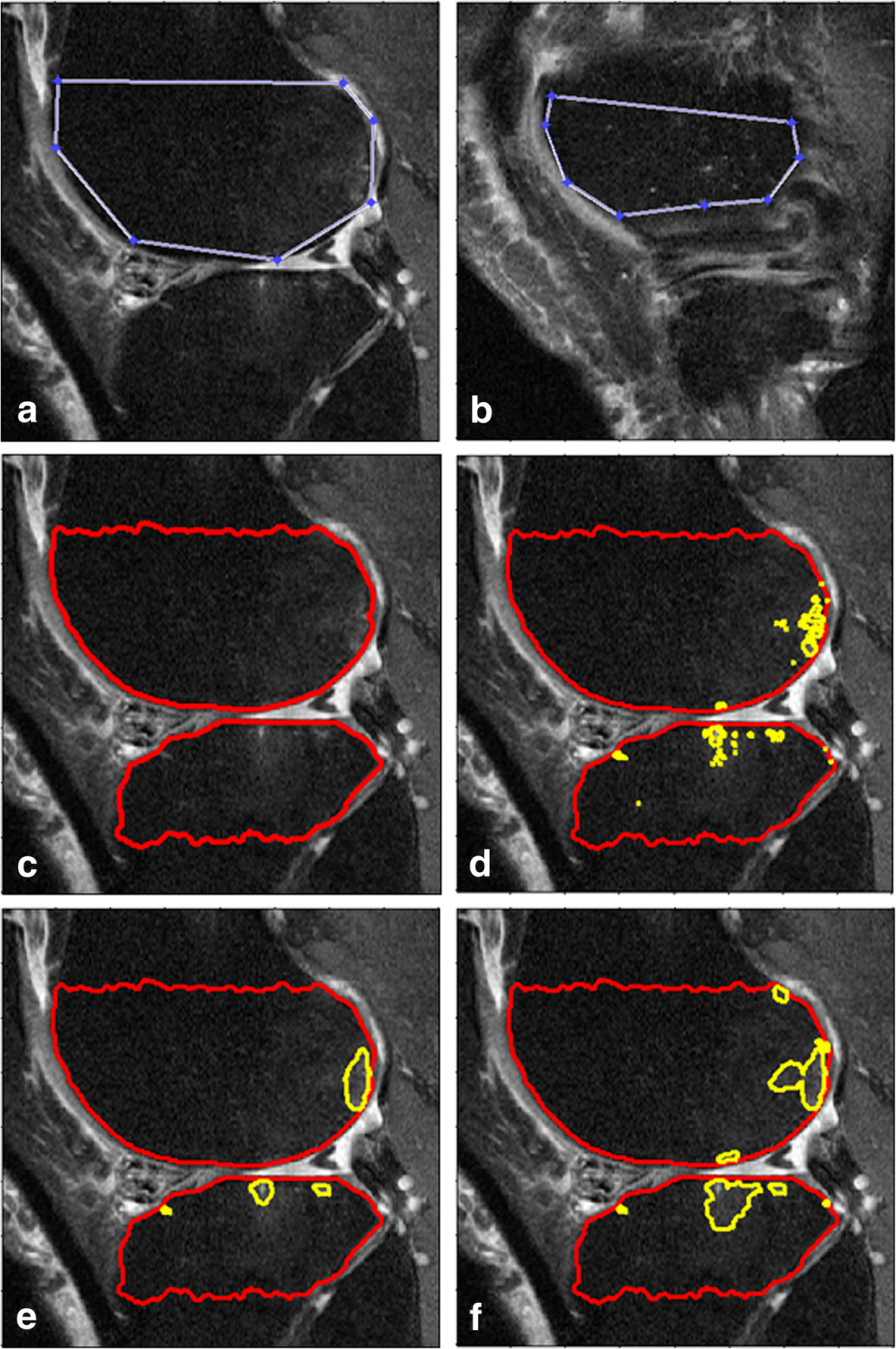
**Example of images from the bone marrow lesion (BML) segmentation process. (a)** Manually marked bone border on a typical slice, **(b)** manually marked bone border on a slice near the edge of the bones, **(c)** the program’s segmentation of the bone, **(d)** the segmentation results just based on thresholding, **(e)** the intermediate segmentation results with bone (red line) and BML (yellow line) by just using one time of thresholding- curve evolution process, **(f)** the final image with bone (red line) and BML (yellow line) segmented.

In the first step we used a custom graphical user interface (GUI; MATLAB, MathWorks, Inc., Natick, MA, USA) to manually identify the boundaries of the tibia and femur in each slice of the MR imaging data set by marking multiple points along the articular surface (see Figure 1a). For the border furthest from the articular surface the rater either marked the bone just prior to the epiphyseal line or at the edge of bone and soft tissue (images near the medial or lateral sides of the knee; Figure 1b). In addition, we omitted the central slices from the analyses (i.e., the middle 9 slices; 2.7 cm) to focus on BMLs adjacent to the tibiofemoral chondral surface and to improve reliability. Excluding these images improved reliability because the segmentation method was inconsistent at defining the border of the bone and there was increased signal-intensity heterogeneity within the bone. Manually marking the bone, the only part of this processing stage involving user interaction, required 8 to 24 min per pair of MR image sets (one knee at two time points) compared to approximately 30 to 45 min per pair of MR image sets using cross-sectional measurements to approximate BML volume [6]. After the tibia and femur boundaries were marked we used an edge-based curve evolution technique [19] to refine the initial estimate to more precisely identify the bone boundaries. An edge-based curve evolution technique [19] was considered optimal because bone typically has well defined boundaries. This process of refinement does not require the initial curves to be immediately adjacent to the bone boundaries (Figure 1a and c) [19].

The second step, BML segmentation, automatically identified the boundaries of the BMLs within the bone region found in the first step by using a region-based curve evolution algorithm [20], which was considered an optimal algorithm because BMLs often have ill-defined boundaries. To segment the BMLs we needed to predefine two parameters (false discovery rate [FDR] [21] and length penalty parameter [μ] [20]). The FDR is the expectation of the ratio of false positives to all significant hypotheses. We used the FDR to determine a threshold between bright area and normal area within the bone region. A greater FDR leads to a lower threshold and therefore more pixels would be identified as a BML. The length penalty parameter (μ; usually between 0 and 1) was used to control the curvature of the segmented BML boundaries. A greater μ leads to smoother the curves and the more frequently relatively small areas of discrete bright pixels would be eliminated. We set FDR = 0.05 and μ = 0.28 in all of our experiments throughout this paper. We tuned up FDR and μ based on the consistency in the results of 10 pairs of knee data.

Based on the FDR, a rough segmentation was obtained for the BMLs by a thresholding approach applied to the MR images (Figure 1d). From the thresholded binary image, we constructed initial curves of the BML boundaries and then a region-based curve evolution method [20] refined the initial curves to obtain an accurate BML border (Figure 1e). In order to capture BMLs whose contrasts vary considerably, we performed the thresholding and curve evolution process a second time while excluding the segmented BMLs identified during the first curve evolution step. Finally, we used the combination of those two BMLs sets as the final BML volumes (Figure 1f). The volumetric measurements of specific BMLs, based on this segmentation approach, have been reported to have a good correlation and agreement with manual measurements of approximate BML volume [6].

The third step of this method, BML refinement and quantification, automatically excluded false positive regions (e.g., connective tissue, imaging artifacts) and measure BML size. Currently we are using two criterions to eliminate the false positive regions: (1) the distance between a BML to the articular surface should be no more than 10 mm [6,14,22] and (2) a BML should span more than one MR image. Finally, we stacked the processed 2-dimensional MR images into one 3D stack data and used criterions above to exclude the false positive regions and obtain the BML volumes. BML volumes are calculated for four discrete regions: medial femur, lateral femur, medial tibia, and lateral tibia.

Intra-tester and inter-tester reliability were assessed for two investigators (JP and GD). To assess intra-tester reliability the investigators segmented 10 (JP) or 12 (GD) knees (baseline and follow-up) from the progression subcohort of the OAI (had symptomatic OA in at least one knee). The investigators repeated their segmentations at least 72 h after performing the initial segmentations. After independently verifying their intra-rater reliability they were provided a data set of 20 knees from the OAI progression subcohort. Each investigator independently segmented the knees. Intra-tester reliability for BML change was good to excellent for investigator one (JP; ICC [3,1 model] = 0.79 to > 0.99) and investigator two (GD; ICC [3,1 model] = 0.95 to 0.96). Inter-tester reliability for BML volume change was good for the lateral femur and tibia as well as the medial femur (ICC [2,1 model] = 0.83 to 0.93) but low for the medial tibia BML volume change (ICC [2,1 model] = 0.59).

### Study 1: comparison to semi-quantitative BML size

We compared data generated by the semi-automated BML segmentation method to Boston Leeds Osteoarthritis Knee Scores (BLOKS) [10] for BMLs (semi-quantitative grade 0 to 3) to assess the construct validity of the new outcome measure. We selected a convenience sample of 80 right knees with area of denuded cartilage from a subset of 160 participants that were members of the progression subcohort (had symptomatic OA in at least one knee) and had acceptable quality fixed-flexion knee radiograph and MR imaging sequences (identified as read project 4 in the public OAI data files entitled kmri_qcart_ecksteinXX [version 0.4 and 3.3]). This sample was convenient because there is extensive image assessment data publicly available among these participants and BLOKS scores were previously assessed by one investigator (GH; weighted kappa = 0.88 [1]). We selected BLOKS scores, instead of other BML scoring methods, based on our experience with BLOKS scoring and prior research which indicated that BLOKS was comparable with Whole Organ MR Scoring (WORMS) method in cross-sectional studies [23].

One investigator (JP) performed the segmentation using the new approach among 80 knees using OAI baseline sagittal intermediate weighted, turbo spine echo, fat-suppressed MR sequences [18]. The variables of interest were medial and lateral tibia BML volume as well as BLOKS BML scores in the medial and lateral tibia. Analyses were restricted to the tibia since the BLOKS system provides a score for the entire medial or lateral tibia region, similar to the region of interest for the segmentation method. Since only a small number of BMLs were detected in the lateral tibia we collapsed medial and lateral tibia for analyses and considered each region to be independently assessed. Therefore, we performed 3 Wilcoxon signed-rank tests to determine if tibia BML volume was significantly different between adjacent BLOKS BML scores (e.g., 1 versus 2, 2 versus 3). Based on these analyses we reported the z statistics and p-values with statistical significance defined as a p-value ≤ 0.05. We did not pursue ordinal logistic regression because the models had significant score test for the proportional odds assumption.

### Study 2: association between BML size and cartilage morphology

We evaluated whether data from the new semi-automated BML segmentation method would replicate findings from previous studies that documented an association between BMLs and cartilage parameters [6,10,24-26]. We selected a convenience sample of 40 knees from 196 knees in the baseline and 24-month OAI visit datasets (kmri_qcart_eckstein [version 0.4 and 3.3]) that had full thickness cartilage loss on the tibia and femur in the index compartment (defined as the tibiofemoral compartment with greater denuded area). In order to have a heterogeneous sample, 20 knees were selected from the medial tibiofemoral index compartment and 20 knees were selected from the lateral tibiofemoral index compartment: knees that had the least change in femur denuded area (*n* = 5), greatest change in femur denuded area (*n* = 5), the least change in tibia denuded area (*n* = 5), and greatest change in tibia denuded area (*n* = 5). The sample size was selected based on the strength of significant correlations we observed in our previous study that explored an association between BML volume and cartilage parameters (*r* = 0.48 to 0.63) [6].

One investigator (JP) performed the segmentation using our new method on the 40 knees using baseline and 24-month sagittal intermediate weighted, turbo spine echo, fat-suppressed MR sequences. The variables of interest were the change in tibia and femur BML volume as well as change in tibia and central femur cartilage thickness and denuded area. The central region of the femur was defined as 60% of the distance between trochlear notch and posterior edge of the femoral cartilage. All of the analyses were limited to the index compartment. We calculated Spearman correlation coefficients to assess the relationship between 2-year BML volume change and 2-year cartilage morphometry change. A significant association was defined by a p-value ≤ 0.05. Potential outliers were explored based on a 95% prediction eclipse. We did not adjust these analyses since the goal was to replicate previously reported associations between two measurements to determine the construct validity of the new BML segmentation method.

## Results

### Comparison to semi-quantitative BML size

**Figure 2:**
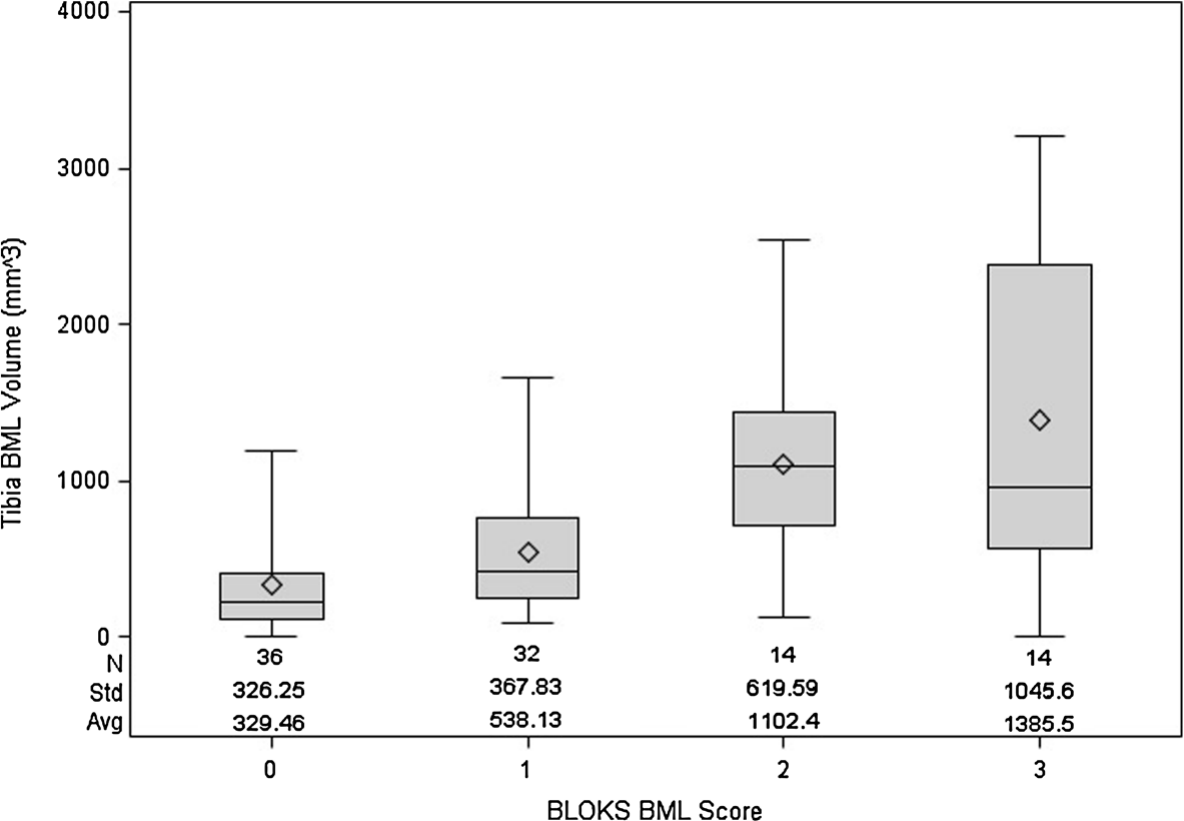
**Box plots showing the distribution of tibia BML volume across Boston Leeds Osteoarthritis Knee Scores (BLOKS).** Each knee contributed two regions (medial and lateral tibia) which were considered to be independently assessed. Boxes represent interquartile range and whiskers represent minimum and maximum medial tibia BML volume.

Among the 80 right knees 48 knees had BLOKS BML scores. In the medial tibia, most knees had no to medium size BMLs based on BLOKS: 11 (23%) knees with BML = 0, 19 (40%) knees with BML = 1, 10 (21%) knees with BML = 2, 8 (17%) knees with BML = 3. In the lateral tibia, most knees had no to medium size BMLs based on BLOKS: 25 (52%) knees with BML = 0, 13 (27%) knees with BML = 1, 4 (8%) knees with BML = 2, 6 (13%) knees with BML = 3. Figure 2 shows the distribution of tibia BML volume across BLOKS BML scores. Overall, the tibial BML volume ranged from 0 mm^3^ to 3204.07 mm^3^ (only 2 knees had BML volume = 0 mm^3^). BML volume was significantly greater among knees with BLOKS BML score = 1 compared to knees with BLOKS BML score = 0 (*z* = 2.85, *p* = 0.004), and among knees with BLOKS BML scores = 2 compared to knees with BLOKS BML score = 1 (*z* = 3.09, *p* = 0.002). There was no significant difference between BLOKS BML scores 2 and 3 (*z* = −0.30, *p* = 0.77).

### Association between BML size and cartilage morphology

We evaluated the association between 2-year change in BML volume and change in cartilage morphometry among 38 knees with denuded area (1 knee was excluded because of poor image quality and another was classified as having collapsed post avascular necrosis). Table 1 provides an overview of the sample characteristics for these analyses.

**Table 1:** Descriptive characteristics of knees used to examine the association between Bone Marrow Lesion (BML) size and cartilage morphology (n = 38)

**Descriptive Characteristic**	Median (Min, Max)* or n (%)
Females	25 (66%)
OAI Progression Subcohort	36 (95%)
Radiographic Knee Osteoarthritis (Kellgren-Lawrence Grade ≥ 2)	38 (100%)
Age (years, mean ± standard deviation)	61 ± 8
Body Mass Index (kg/m^2^; mean ± standard deviation)	29.9 ± 5.3
Tibia BML Volume (baseline; mm^3^)	643.88 (17.97, 8120.29)
Femur BML Volume (baseline; mm^3^)	971.55 (21.79, 4205.43)
Tibia BML Volume Change (mm^3^)	86.22 (−5824.68, 3050.37)
Femur BML Volume Change (mm^3^)	164.41 (2268.4, 4634.04)
Tibia Cartilage Thickness Change (mm)	−0.10 (−0.54, 0.19)
Central Femur Cartilage Change (mm)	−0.18 (−0.96, 0.26)
Tibia Denuded Area Change (%)	3.9 (−9.4, 36.2)
Central Femur Denuded Area Change (%)	5.3 (−9.4, 36.2)

* Median (Min, Max) reported instead of mean + standard deviation unless noted otherwise.

An increase in tibia BML volume was significantly related to an increase in tibia denuded area (Spearman *r* = 0.42, *p* = 0.008) and a decrease in tibia cartilage thickness (Spearman *r* = −0.46, *p* = 0.004). Furthermore, increases in tibia BML volume was associated with increased femur denuded area (Spearman *r* = 0.35, *p* = 0.03) and possibly decreased femur cartilage thickness (Spearman *r* = −0.30, *p* = 0.07) but this last finding was not statistically significant. No associations to femur BML volume change were statistically significant (Spearman *r* = −0.15 to 0.15).

## Discussion

In this manuscript we described the validity of a novel semi-automated method to quantify BML size. This method has several advantages compared to existing methods. First, our method yields quantitative BML volume unlike manual linear measurements (e.g., greatest diameter [5], approximate BML volume [6,11]) or semi-quantitative scoring methods (e.g., BLOKS [10], WORMS [8]). Second, unlike manual segmentation methods [13,14] our method does not require expert readers since the reader’s primary responsibility is to mark the border of the bone, thus automating the BML segmentation step. There are some other semi-automated methods [16,17] to quantify BML volume, however their methods are based on thresholding while our method performed some refinement on thresholding results by eliminating small isolated bright areas, as well as merging and evolving closed bright areas (See Figure 1d and e). We found that the quantitative measure of BML volume increased across semi-quantitative scoring and related to changes in cartilage thickness and denuded area, confirming previous reports of this association (construct validity).

Compared to BLOKS, the new measure of BML volume increased across grades in the tibia, with the exception of between grades 2 and 3. This suggests that our approach yields consistent results with BLOKS scale system in the evaluation of BMLs. The lack of statistical significance between BLOKS BML scores 2 and 3 in the medial tibia may be related to the available sample size or the semi-quantitative nature of BLOKS BML scores (e.g., potential for misclassifications). While some issues arise from the semi-quantitative readings another issues may be that the BML segmentation algorithm detects any region of high signal intensity within the bone that appears on multiple adjacent images (e.g., bone marrow lesions, subchondral cysts, blood vessels). This limitation led us to hypothesize that the BML segmentation method should be used primarily for detecting longitudinal change in BML volume since static structures will not change over time. Since longitudinal studies of BML size may be the main use of this program the next step was to assess the construct validity of BML volume change.

Our second study agreed with previous reports that change in BML size relates to longitudinal increases in denuded area and decreased cartilage volume [5]. More specifically, we found that increases in tibia BML volume were related to tibia cartilage loss while no associations were detected in the femur. The lack of association between femur BML and femur cartilage loss may be because the cartilage was assessing the central weight-bearing region of the medial femur while the BMLs were assessed throughout the medial femur (e.g., posterior femoral condyle, trochlear region).

Based on these findings, the new semi-automated BML segmentation method appears to be a valid assessment of BML volume, particularly BML volume change. There are several advantages of our method compared to existing methods. Firstly, this new approach can accurately measure volume rather than rough indices (e.g., greatest diameter [5], approximate BML volume [6,11]). In addition to improved accuracy, the semi-automated BML segmentation method offers good intra-rater reliability and adequate inter-rater reliability to allow for multiple raters if a quality assessment step is in place to ensure consistent bone segmentation. This extra quality assessment procedure is not uncommon in other areas of knee segmentation [27]. It is important to note that raters only mark the edges of the bone and therefore raters are not responsible for adjusting the contrast or identifying the boundary of a region that is not well delineated. The boundary of unclear BMLs would be located by the active contour driven by pixel intensity distribution automatically. Our approach should be more consistent than prior methods because it segments the BMLs from the bone region directly based on the intensity of the MR images without doing any preprocessing.

In addition to good reliability, our approach is a very time efficient method to quantify the BMLs. The current approach requires about 4 to 12 min for raters to initially segment the bones, depending on the number of images. The major reason for improved time efficiency is the reduction of manual operation. Only the first step needs manual operation and the remaining two steps are fully automated and take about 4 to 5 min using Matlab code on a personal computer with an Intel Core 2.66 GHz CPU and 2.0 GB RAM. Moreover, bone boundaries have distinct patterns and are less subjective to segment than BMLs. Based on the inter-rater and intra-rater reliability as well as the reduced manual operation time the semi-automated method can be used to more efficiently investigate larger MR imaging data sets.

While the new semi-automated BML segmentation method has many advantages that make it appealing for implementation there are some limitation and opportunities for improvement. Once the bone initialization (the first step) is finished, various algorithms could be proposed to perform bone segmentation and BML segmentation which might be faster and yield more robust and consistent results. For example, the current algorithm for BML segmentation only uses the mean and standard deviation of the pixel intensity in the MR images, but some other statistical variables of the intensities or even location information of BMLs could be incorporated to improve the segmentation results. In addition, once the segmentation of the bone and the BMLs is finished in a larger data set, other features of BMLs can be investigated besides BML volume. For example, users could extract the distance, intensity distribution, or other features of the BMLs. Moreover, the current algorithm for bone segmentation could be improved by more advanced algorithms. Those more advanced algorithms could further reduce or even omit manual operation which would make our approach more efficient and robust.

Optimizing the algorithms may improve the segmentation results in the central region of the knee, which was excluded in these analyses, as well as help differentiate BMLs and subchondral cysts. This region was omitted because of technical challenges in accurately identifying the border of the bone and increased signal-intensity heterogeneity. Excluding this region may bias the BML results by under representing BMLs associated with patellofemoral joint changes but may be advantageous for evaluating the tibiofemoral joint. Previous research has suggested that centrally-located BMLs do not typically influence tibiofemoral cartilage unless they extend into the medial tibiofemoral compartment [28]. New algorithms may also lead to the ability to discriminate BMLs and subchondral cysts, which could facilitate studies that clarify the etiology of both lesions and how they change over time. In the current analyses, we considered BMLs and subchondral cysts as one classification of subchondral changes since subchondral cysts often develop in regions with BMLs [29,30] and both types of lesions are associated with pain and structural changes [1-7,31,32]. When interpreting results with this BML segmentation method it is important to consider that these results primarily reflect tibiofemoral BMLs that may include cystic changes.

While these validation studies demonstrated the validity of the new semi-automated BML segmentation methods there are some important limitations to these analyses. For example, we compared the new BML volume to BLOKS, a well validated measure of BML size but BLOKS is not a gold standard and does not offer a quantitative comparison. Ideally, the new BML volume would have been compared to another accurate quantitative outcome or histology but these methods also introduce various limitations (e.g., lack of agreement on the histological presentation of BMLs, lack of gold standard quantitative BML measures). Furthermore, the validation studies were all conducted using sagittal intermediate weighted turbo spin echo with fat suppression images collected at the four OAI clinical sites using 3 T Siemens scanners. The BML segmentation approach may need to be reevaluated when deployed in new data sets using different MR scanners, MR sequences, or study populations (e.g., knees without full thickness cartilage loss). The limited generalizability of these validation studies does not limit the usefulness of this new method particularly because any new method would also need to be reevaluated when deployed in a new study protocol or population.

## Conclusions

In summary, we proposed a novel semi-automated method to quantify BML sizes. We validated our new method by demonstrating that the new BML volume measurement increases with greater BLOKS BML scores and confirming previous reports that BML size is associated with longitudinal cartilage loss among knees with full thickness cartilage loss. In addition, our method is time efficient (about 7 min per knee MR scan) and yields adequate intra- and inter-rater reliability to allow multiple raters to process the MR sequences if an additional quality assessment step is performed to help ensure consistency between raters. This new method will enable researchers to assess larger MR data sets in a time efficient manner.

## Abbreviations

BLOKS: Boston Leeds Osteoarthritis Knee Scores; BML: Bone marrow lesion; FDR: False Detection Rate; ICC: Intra-class correlation coefficient; MR: Magnetic resonance; OA: Osteoarthritis; OAI: Osteoarthritis Initiative; μ: Length penalty parameter.

## Competing interests

FE is CEO of Chondrometrics GmbH, a company providing MR image segmentation services. He provides consulting services to MerckSerono, Novartis, Sanofi-Aventis and Perceptive, and has recently received speaking honoraria from Merck, Genzyme, Glaxo Smith Kline, Synthes, and Medtronic. No other authors declare that they have no competing interests.

## Authors’ contributions

JP contributed to the conception and design, acquisition of data, analysis and interpretation of data, drafting/revisions of article, as well as final approval of the article. JBD contributed to the conception and design, acquisition of data, analysis and interpretation of data, drafting/revisions of article, as well as final approval of the article. GD contributed to the acquisition of data, drafting/revisions of article, as well as final approval of the article. EM contributed to the conception and design, analysis and interpretation of data, drafting/revisions of article, as well as final approval of the article. GHL contributed to the conception and design, analysis and interpretation of data, drafting/revisions of article, as well as final approval of the article. RJW contributed to the conception and design, analysis and interpretation of data, drafting/revisions of article, as well as final approval of the article. LLP contributed to the conception and design, analysis and interpretation of data, drafting/revisions of article, as well as final approval of the article. JAL contributed to the acquisition of data, interpretation of data, drafting/revisions of article, as well as final approval of the article. CBE contributed to the acquisition of data, interpretation of data, drafting/revisions of article, as well as final approval of the article. FE contributed to the acquisition of data, interpretation of data, as well as final approval of the article. TEM contributed to the conception and design, analysis and interpretation of data, drafting/revisions of article, as well as final approval of the article. All authors read and approved the final manuscript.

## Pre-publication history

The pre-publication history for this paper can be accessed here:

http://www.biomedcentral.com/1471-2474/14/3/prepub
